# Superresolution characterization of core centriole architecture

**DOI:** 10.1083/jcb.202005103

**Published:** 2021-02-03

**Authors:** Yuan Tian, Chenxi Wei, Jianfeng He, Yuxuan Yan, Nan Pang, Xiaomin Fang, Xin Liang, Jingyan Fu

**Affiliations:** 1 State Key Laboratory of Agrobiotechnology, College of Biological Sciences, China Agricultural University, Beijing, China; 2 Tsinghua-Peking Joint Center for Life Sciences and Max Planck Partner Group, School of Life Sciences, Tsinghua University, Beijing, China

## Abstract

The centrosome is the main microtubule-organizing center in animal cells. It comprises of two centrioles and the surrounding pericentriolar material. Protein organization at the outer layer of the centriole and outward has been studied extensively; however, an overall picture of the protein architecture at the centriole core has been missing. Here we report a direct view of *Drosophila* centriolar proteins at ∼50-nm resolution. This reveals a Sas6 ring at the C-terminus, where it overlaps with the C-terminus of Cep135. The ninefold symmetrical pattern of Cep135 is further conveyed through Ana1–Asterless axes that extend past the microtubule wall from between the blades. Ana3 and Rcd4, whose termini are close to Cep135, are arranged in ninefold symmetry that does not match the above axes. During centriole biogenesis, Ana3 and Rcd4 are sequentially loaded on the newly formed centriole and are required for centriole-to-centrosome conversion through recruiting the Cep135–Ana1–Asterless complex. Together, our results provide a spatiotemporal map of the centriole core and implications of how the structure might be built.

## Teaser

Proteins within the core region of the centriole remain challenging to be resolved in vivo. Tian et al. present a spatiotemporal map of the centriole core and implications of how the proteins might interact to build the ninefold symmetrical centriole.

## Introduction

The centrosome has multiple crucial functions, including the assembly of the mitotic spindle and establishing the axis of cell division. It comprises two principal components: a pair of orthogonally arranged centrioles and the surrounding pericentriolar material (PCM). Centrioles are stable cylindrical structures comprising nine microtubule blades arranged at the end of nine spokes that radiate from a central hub. During each cell cycle, the centriole pair disengages at the mitotic exit, allowing the new centrioles (or daughter centrioles) to gradually assemble next to each preexisting centriole (the mother centriole). A mother centriole servesas a recruitment and assembly scaffold for the PCM proteins to form spindle poles in mitosis; in many cell types, it also provides a template for cilium or flagellum assembly during cell quiescence, forming a crucial organelle for chemical sensation, signal transduction, locomotion, and so forth. Centrosome defects have been related to a wide range of human diseases, including cancer, microcephaly, and a group of disorders collectively known as the “ciliopathies” (Breslow and Holland, 2019; Chavali et al., 2014; Fu et al., 2015; Godinho and Pellman, 2014; Nigg and Holland, 2018).

**Figure 1.**
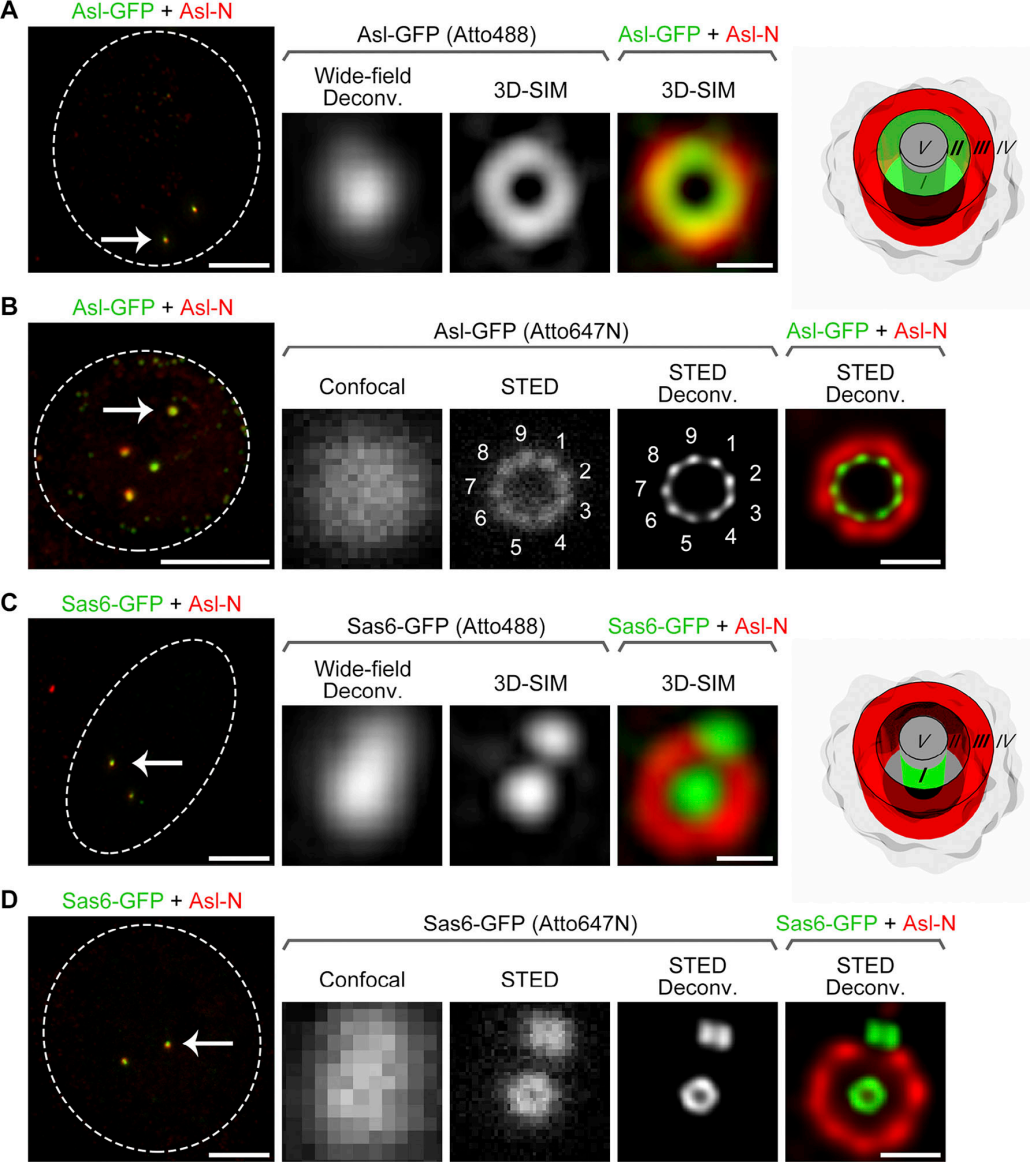
**Direct visualization of ninefold symmetry at Asl C-terminus and a ring at Sas6 C-terminus. (A)** D.Mel-2 cells constitutively expressing Asl-GFP were immunostained with GFP-booster Atto488 (green) and antibody against the N-terminus of Asl (Asl-N; mother centriole marker, red) and analyzed by 3D-SIM. The GFP signal at the Asl C-terminus was revealed as a ring at zone II, and the signal of Asl-N was at zone III. Left panel presents the whole cell; the dashed line indicates the cell border; and the arrow marks the centrosome that is zoomed in the right panels. Bar for cell, 5 µm; for zoomed centrosome, 200 nm. Wide-field Deconv., deconvolution of the 3D-SIM raw data; 3D-SIM, reconstruction of the same raw data (superresolution). **(B)** D.Mel-2 cells constitutively expressing Asl-GFP were immunostained with GFP-booster Atto647N (green) and antibody against Asl-N (red) and analyzed by STED microscopy. Note that the GFP signal at Asl C-terminus was resolved into ninefold symmetrical densities in both raw data (STED) and a deconvolved image (STED Deconv.). Left panel presents the whole cell; the dashed line indicates the cell border; and the arrow marks the centrosome that is zoomed in the right panels. Bar for cell, 5 µm; for zoomed centrosome, 200 nm. **(C)** D.Mel-2 cells constitutively expressing Sas6-GFP were treated as in A. GFP signal at the Sas6 C-terminus was revealed as a dot at zone I by 3D-SIM. Left panel presents the whole cell; the dashed line indicates the cell border; and the arrow marks the centrosome that is zoomed in the right panels. Bar for cell, 5 µm; for zoomed centrosome, 200 nm. **(D)** D.Mel-2 cells constitutively expressing Sas6-GFP were treated as in B. GFP signal at Sas6 C-terminus was resolved into a ring by STED microscopy. Left panel presents the whole cell; the dashed line indicates the cell border; and the arrow marks the centrosome that is zoomed in the right panels. Bar for cell, 5 µm; for zoomed centrosome, 200 nm.

Understanding how the centrosome functions requires knowledge of its protein composition and organization. The centrosome is composed of >100 different proteins (Andersen et al., 2003; Jakobsen et al., 2011; Müller et al., 2010). Their architectural arrangement has begun to be systematically examined since the application of superresolution microscopy (Fu and Zhang, 2019). Using 3D structured illumination microscopy (3D-SIM), our and others’ work revealed distinct concentric domains within a centrosome (e.g., zones I–V of the *Drosophila* centrosome; Fig. 1, A and C) and that the PCM has a conserved, ordered structure (Fu and Glover, 2012; Lawo et al., 2012; Mennella et al., 2012; Sonnen et al., 2012). Protein organization at several compartments of the centrosome, such as the distal and subdistal appendages, the transition zone, the centrosome linker, and the longitudinal axis of the centriole, has also been studied via 3D-SIM (Huang et al., 2017; Lee et al., 2014; Sir et al., 2011; Sydor et al., 2018), stimulated emission depletion (STED) microscopy (Lau et al., 2012; Lee et al., 2014; Lukinavičius et al., 2013; Vlijm et al., 2018; Yang et al., 2015), or stochastic optical reconstruction microscopy (Bowler et al., 2019; Shi et al., 2017; Sillibourne et al., 2011; Yang et al., 2018). Meanwhile, proteins at the core of the centriole remain largely unresolved. This cartwheel region, revealed as zone I by 3D-SIM (Fu and Glover, 2012), contains the central hub of ∼22-nm diameter and the nine spokes that determine the ninefold symmetrical feature of the centriole (Guichard et al., 2012; Guichard et al., 2017).

*Drosophila* cultured cells present a consistent model for the study of the centriole core because, contrary to the vertebrate centrosome, the cartwheel persists in the mature centriole (Debec and Marcaillou, 1997; Dzhindzhev et al., 2014). The centriole is composed of doublet microtubules arranged in a ninefold symmetrical cylinder, which is ∼200 nm wide and long and has a cartwheel formation along the entire length (Callaini et al., 1997; Debec and Marcaillou, 1997; Debec et al., 1999; Lattao et al., 2017). In this study, we first determined which proteins known to be required for *Drosophila* centriole duplication (Dobbelaere et al., 2008; Goshima et al., 2007) are the components of the centriole core. We then present a direct view of these proteins at ∼50-nm resolution and a timing order of their assembly using several superresolution techniques. These revealed a ninefold radial scaffold comprising Spindle assembly abnormal 6 (Sas6), Centrosomal protein 135kDa (Cep135), Anastral spindle 1 (Ana1), and Asterless (Asl), as well as concentric toroids formed by Anastral spindle 3 (Ana3) and Reduction in Cnn dots 4 (Rcd4), two novel core centriolar components that are also organized in ninefold symmetry. During centriole biogenesis, Ana3 is recruited to the newly formed daughter centriole later than Sas6 but before Rcd4 and Cep135. Our findings thus provide a spatiotemporal map of the centriole core and a model of how the proteins might interact to build the structure.

## Results

### Direct visualization of ninefold symmetrical distribution of Asl

**Figure S1.**
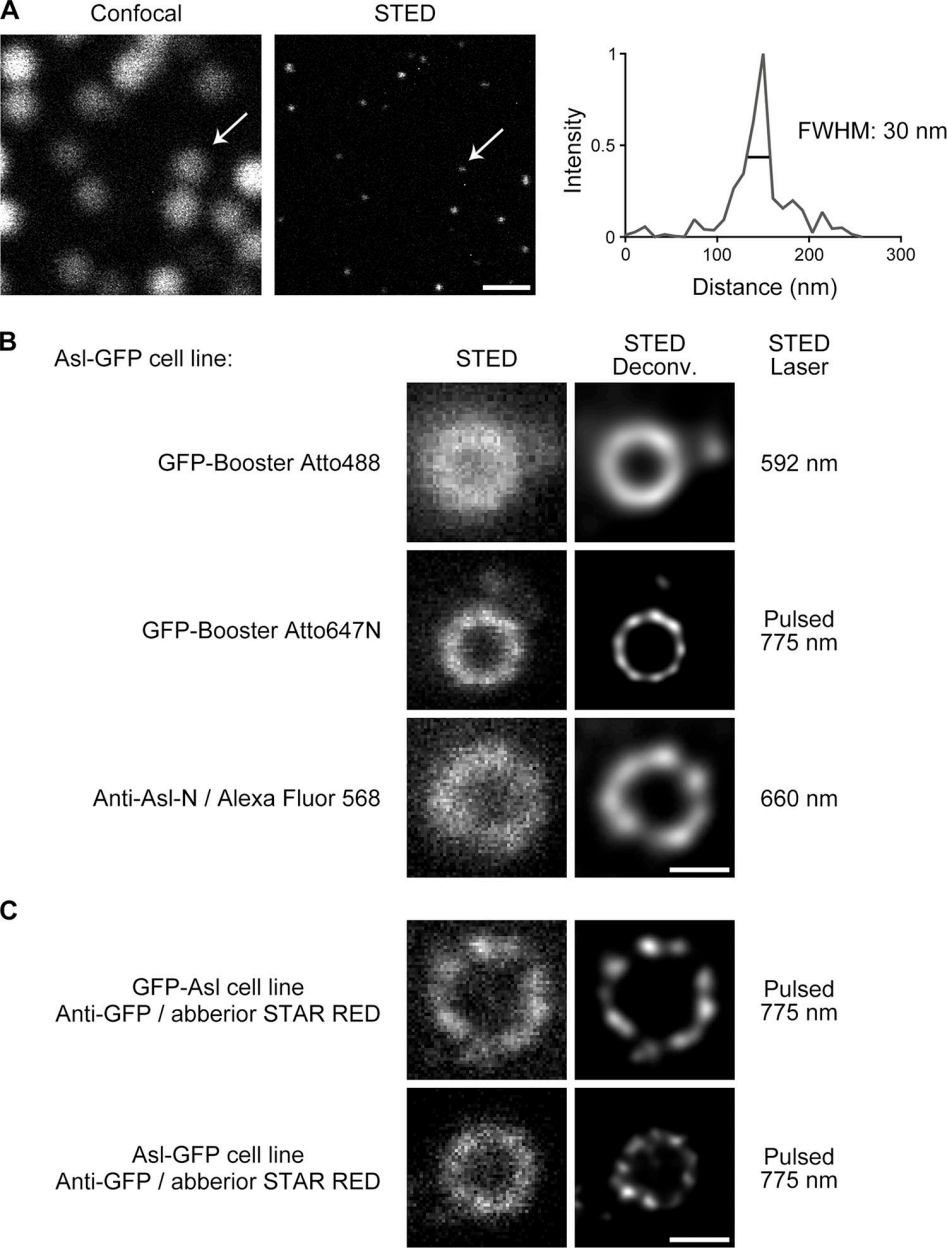
**Pulsed STED laser and time-gated detection render better resolution. (A)** Comparison of confocal and STED raw images of 40-nm red fluorescent nanoparticles. STED image was taken using pulsed STED laser at 775 nm. Arrows indicate one representative nanoparticle whose STED profile is on the right. The average full width at half maximum (FWHM) is measured as 35 ± 7 nm and *n* = 33. Bar, 500 nm. **(B)** D.Mel-2 cells constitutively expressing Asl-GFP were immunostained with GFP-booster Atto488, GFP-booster Atto647N, or primary antibody against the N-terminus of Asl (Anti-Asl-N) and secondary antibody conjugated with Alexa Fluor 568. Note that the pulsed STED laser at 775 nm (targeting Atto647N) renders better resolution than the other two laser lines at 592 nm (targeting Atto488) and 660 nm (targeting Alexa Fluor 568). Bar, 200 nm. **(C)** D.Mel-2 cells constitutively expressing GFP-Asl or Asl-GFP were immunostained with GFP antibody and secondary antibody conjugated with Abberior STAR RED. Both N and C termini of Asl are organized as nine discrete signals resolved by pulsed STED laser 775 nm. Bar, 200 nm.

**Figure S2.**
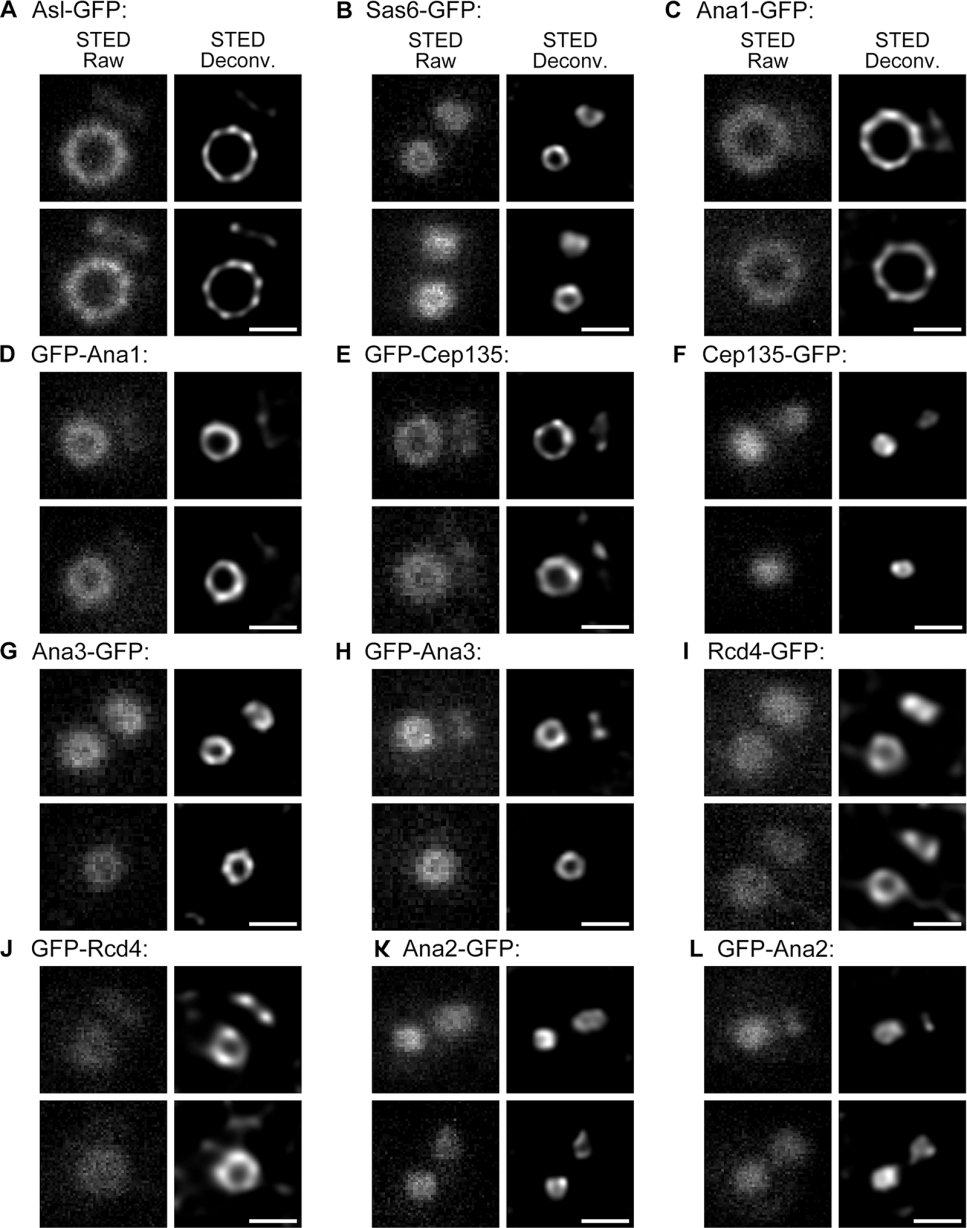
**Representative STED images of centriolar proteins.** **(A–L)** D.Mel-2 cells constitutively expressing indicated GFP-tagged protein were immunostained with GFP-booster Atto647N and antibody against the N-terminus of Asl (mother centriole marker, not shown) and analyzed by STED microscopy. Raw data are shown in left panels and deconvolved images in the right. Bars, 200 nm.

To gain insight into how proteins at the centriole core are organized in the native state, we applied pulsed, gated STED microscopy mounted with a single-molecule detection detector, a purely optical microscope that yields resolution below 50 nm without image processing (Fig. S1 A). We first tested the system by analyzing *Drosophila* Asl (homologue of human Cep152), which presents a characteristic extended configuration: The C-terminus of Asl, when tagged with GFP, is constantly resolved as a zone II ring of 233 ± 23–nm diameter by 3D-SIM (Fig. 1 A; Fu et al., 2016), whereas the N-terminus tagged with Flag shows a ring signal of 364 ± 32–nm diameter at zone III (Fu et al., 2016). We stained D.Mel-2 cells constitutively expressing Asl-GFP with primary and Alexa Fluor 568–conjugated secondary antibodies to label the N-terminus of Asl and GFP-booster Atto647N nanobody to label GFP. With a pulsed STED laser at 775 nm, a clear ninefold symmetrical distribution was revealed at the C-terminus of Asl in the raw image (Fig. 1 B and Fig. S2 A). Further deconvolution sharpened the symmetrical densities and yielded a higher-resolution representation. The average diameter of Asl C-terminus was 202 ± 6 nm (*n* = 21), consistent with the 3D-SIM data (Fu et al., 2016) but with a much smaller deviation. The N-terminus of Asl was revealed as a ring of larger diameter consistent with 3D-SIM data (Fig. 1, A and B); however, the ninefold symmetry seemed to be less clear, likely due to the lower efficiency of the nonpulsed STED laser at 660 nm (Fig. S1 B). Staining the cells with GFP-booster Atto488 also revealed lower resolution with the nonpulsed STED laser at 592 nm (Fig. S1 B). Thus, the green and red channels were only used for the reference protein (N-terminus of Asl) in the subsequent STED experiments. Meanwhile, staining D.Mel-2 cells constitutively expressing GFP-Asl with primary and Abberior STAR RED–conjugated secondary antibodies to label GFP revealed nine discrete signals (Fig. S1 C), demonstrating that the N-terminus of Asl is organized in ninefold symmetry similar to its Cterminus.

### Direct visualization of a ring at Sas6 C-terminus

**Figure S3.**
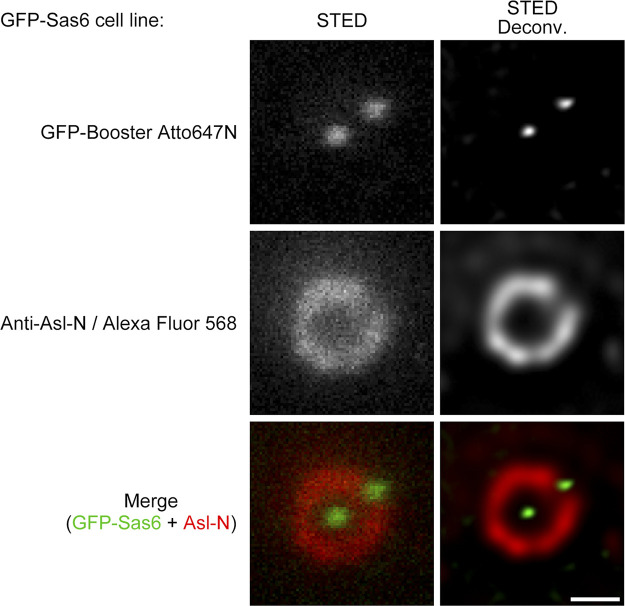
**N-terminus of Sas6 is resolved as a dot by STED microscopy.** D.Mel-2 cells constitutively expressing GFP-Sas6 were immunostained with GFP-booster Atto647N (green) and antibody against the N-terminus of Asl (Anti-Asl-N; mother centriole marker, red). The N-terminus of Sas6 was resolved as a dot in either raw or deconvolved STED images. Bar, 200 nm.

We next analyzed Sas6, the innermost centriolar protein known so far and suggested to be the building block of the ninefold symmetrical cartwheel (Kitagawa et al., 2011; van Breugel et al., 2011). 3D-SIM resolved it as a dot in zone I (Fig. 1 C; Fu and Glover, 2012; Fu et al., 2016). A combined 3D-SIM/single-molecule localization microscopy approach that collects >1,000 protein localizations from many centrioles to calculate a mean localization has achieved higher resolution; yet, as the authors stated, it tends to overestimate small radial distances (Gartenmann et al., 2017). Here, STED immediately resolved Sas6-GFP as a small ring without image processing (Fig. 1 D and Fig. S2 B), providing accurate localization information for the GFP signal at the C-terminus of Sas6. The average diameter was 73 ± 4 nm (*n* = 20) without considering the size of the GFP nanobody, which is 2 nm and barely affects the result. The N-terminus of Sas6, reported from the in vitro studies to form the central hub of ∼22-nm diameter (Guichard et al., 2012; Guichard et al., 2017), remained unresolvable in vivo (Fig. S3).

### Cep135–Ana1–Asl axes are organized in ninefold manner overlapping with Sas6

We sought to ask how other core centriolar proteins are organized together. Previous genome-wide RNAi screens have revealed 18 centrosomal proteins that are required for *Drosophila* centriole duplication (Dobbelaere et al., 2008; Goshima et al., 2007), and some have been well studied but some have not. We tagged these proteins with GFP at either the N- or C-terminus, established stable cell lines, and performed STED microscopy on the fixed cell populations. In addition to Sas6, Anastral spindle 2 (Ana2), Cep135, and Ana1 that have been reported to localize inside the microtubule wall, we found two lesser-known centrosomal proteins at this region: Ana3 and Rcd4.

**Figure 2.**
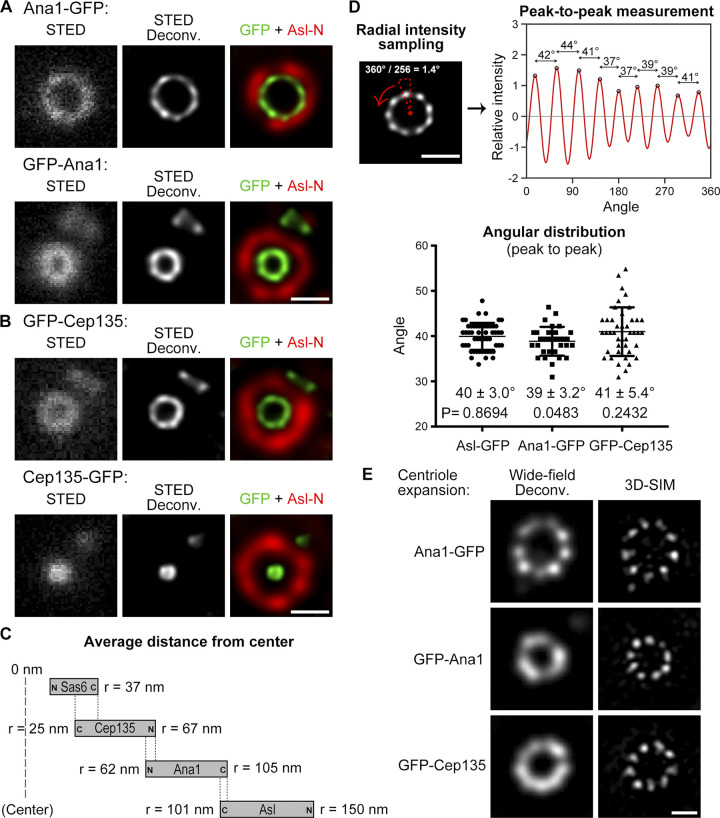
**Cep135–Ana1–Asl axes are organized in ninefold manner and overlap with Sas6. (A and B)** D.Mel-2 cells constitutively expressing GFP-tagged Ana1 (A) or Cep135 (B) were immunostained with GFP-booster Atto647N (green) and antibody against the N-terminus of Asl (Asl-N; mother centriole marker, red) and analyzed by STED microscopy. Bars, 200 nm. **(C)** Schematics showing the relative positions of Sas6, Cep135, Ana1, and Asl within a single centriole. r indicates the average distance between the protein terminus (each tagged with GFP and stained with GFP-booster Atto647N) and the center of the centriole. **(D)** The angular distributions of peak-to-peak intensities from Asl-GFP, Ana1-GFP, and GFP-Cep135 toroids. Upper panels show data of a single centriole taken for illustration purposes. 360° of the centriole are equally divided into 256 angles, and intensities within each sector (dotted triangle; radial intensities) are measured and plotted. The distance between neighboring peaks that corresponds to an angular value is determined. Bar, 200 nm. Lower panel presents the overall data; and left to right, *n* = 53, 33, and 42 peaks. The mean angle ± SD (error bars) and the P value are shown under each plot; a two-tailed one-sample Student’s *t* test was performed with null hypothesis angle = 40°. Note that the angular distributions are consistent with ninefold symmetry, corresponding to a 40° angle. **(E)** D.Mel-2 cells constitutively expressing Ana1-GFP, GFP-Ana1, or GFP-Cep135 were treated using the U-ExM protocol, immunostained with Asl (mother centriole marker, not shown) and GFP antibodies, and analyzed by 3D-SIM. Note that centrioles are physically expanded 4- to 4.5-fold. The ninefold symmetry of Ana1-GFP can be resolved by either deconvolution or reconstruction of the 3D-SIM raw data (Wide-field Deconv. and 3D-SIM, respectively), whereas that of GFP-Ana1 and GFP-Cep135 can only be resolved in reconstructed images. Bar, 500 nm.

The above proteins could be immediately divided into two groups: elongated molecules whose N- and C-termini were far apart and compact molecules that did not show much distance between the two termini. We previously showed that Cep135, Ana1, and Asl are elongated molecules forming a scaffold from zones I to III (Fu and Glover, 2016; Fu et al., 2016). STED again confirmed the observation with higher resolution (Fig. 2, A–C; and Fig. S2, C–F); more important, all three proteins were found to be organized in a ninefold manner (Fig. 2 D), indicating that they are the bona fide components of the spoke–pinhead scaffold and transmit the ninefold symmetrical geometry from the central hub to the outer region as we previously suggested (Fu and Glover, 2016; Fu et al., 2016). Because the ninefold organization of Ana1-GFP, GFP-Ana1, and GFP-Cep135 signals were not always obvious by eyesight alone, we performed ultrastructure expansion microscopy (U-ExM) by which the centriole was physically expanded 4- to 4.5-fold (Gambarotto et al., 2019) and then performed imaging by using 3D-SIM (Fig. 2 E). The ninefold symmetrical distribution of Ana1-GFP could be readily seen in deconvolved images from the 3D-SIM raw data (widefield deconvolved image), whereas GFP-Ana1 and GFP-Cep135 were resolved into nine discrete dots after reconstruction of the raw data (3D-SIM image). In addition, Sas6 formed an elongated conformation overlapping with the C-terminus of Cep135 (Fig. 2 C), the innermost region of the Cep135–Ana1–Asl axes. This indicates a potential interaction between Sas6 and Cep135, which is conserved to their human counterparts (Lin et al., 2013) and provides an explanation for how *Chlamydomonas* Sas6 and the C-terminus of Cep135 could better assemble into a cartwheel-like structure in vitro (Guichard et al., 2017).

### Cep135–Ana1–Asl axes extend past the microtubule blades

**Figure S4.**
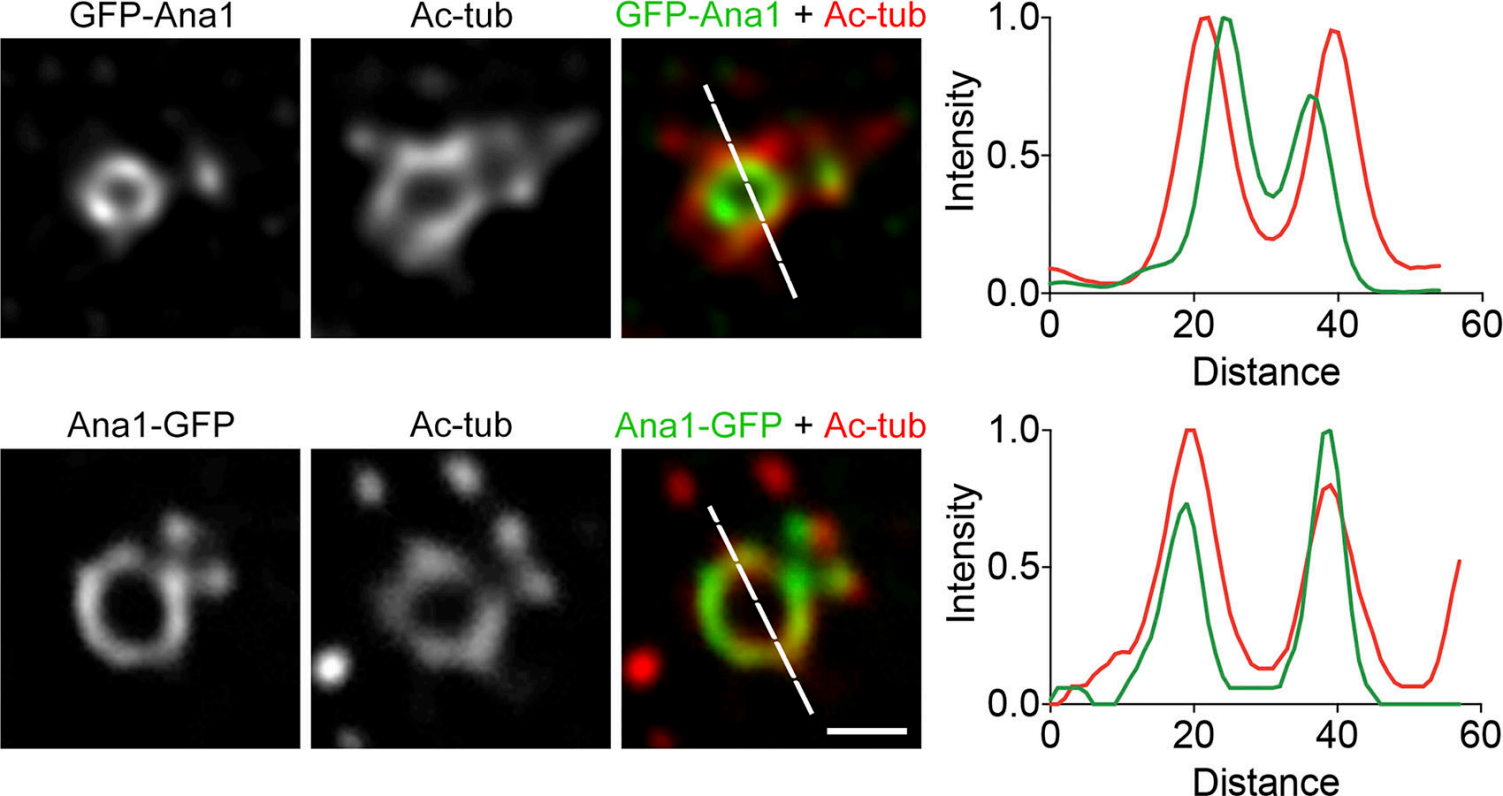
**C-terminus of Ana1 colocalizes with microtubule wall.** D.Mel-2 cells constitutively expressing GFP-Ana1 or Ana1-GFP were treated with colchicine to depolymerize the cytoplasmic microtubules, immunostained with GFP-booster Atto488 (green) and acetylated tubulin antibody (Ac-tub, red), and analyzed by STED microscopy. Note that acetylated tubulin signal colocalizes with Ana1-GFP while significantly outward compared with the GFP-Ana1 signal. Bar, 200 nm.

We next asked whether the ninefold axes could pass through the centriole microtubule wall. We costained cell lines constitutively expressing GFP-Ana1 or Ana1-GFP with the antibody against acetylated tubulin. To this end, we switched to GFP-booster Atto488 and Alexa Fluor 568–conjugated secondary antibody to gain similar resolution for both channels (Fig. S4). We found that the N-terminus of Ana1 was localized inside the microtubule wall, whereas its C-terminus highly colocalized with acetylated tubulin, suggesting that Ana1 extends past the microtubule wall via its C-terminus.

**Figure 3.**
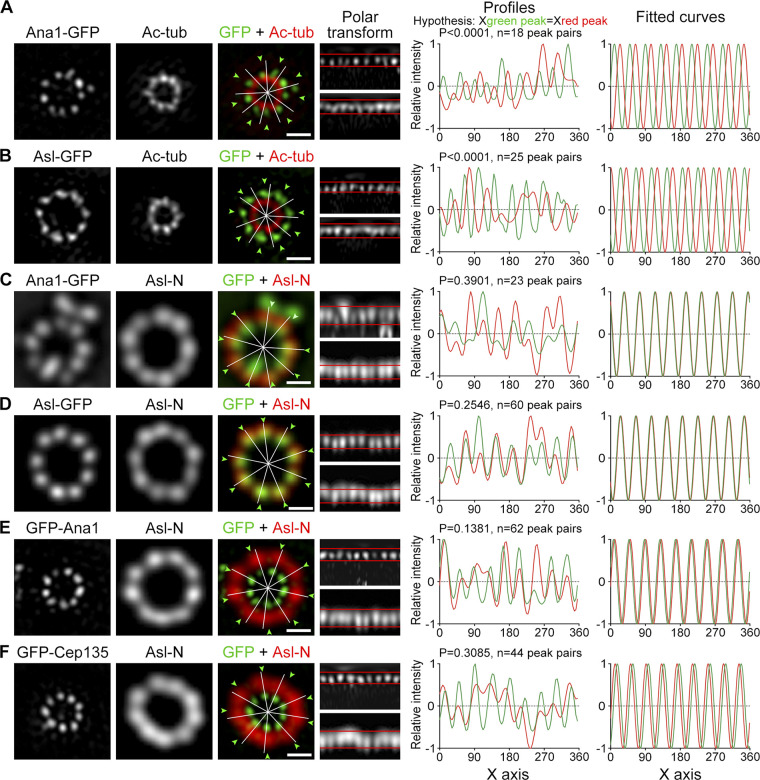
**Cep135–Ana1–Asl axes extend past the microtubule blades. (A and B)** D.Mel-2 cells constitutively expressing Ana1-GFP (A) and Asl-GFP (B) were treated with colchicine to depolymerize the cytoplasmic microtubules, processed with the U-ExM protocol, immunostained with GFP (green) and acetylated tubulin (Ac-tub, red) antibodies, and imaged by 3D-SIM. Reconstructed 3D-SIM images were used for data analysis. Signals of the toroids were transformed to polar coordinates (polar transform, upper band for green channel and lower for red); the intensity profiles were plotted; and the x coordinate of every green peak was compared with that of the corresponding red peak by using a paired, two-tailed Student’s *t* test (hypothesis: X_green peak_ = X_red peak_, and n indicates the number of peak pairs). Both P values are <0.0001, suggesting that the ninefold symmetry of Ana1-GFP and Asl-GFP does not match that of Ac-tub. The peak intensities were also indicated in the original toroids (white lines for red signals and arrowheads for green), and in the right panel, the intensity profiles were fitted to sine curves. Bars, 500 nm. **(C–F)** D.Mel-2 cells constitutively expressing indicated GFP-tagged protein were treated using the U-ExM protocol, immunostained with GFP (green) and the N-terminus of Asl (Asl-N, recognizes 1–300 aa, red) antibodies, and imaged by 3D-SIM. Deconvolved images are used to analyze proteins with large diameters (Asl-N, Ana1-GFP, and Asl-GFP) and reconstructed 3D-SIM images for proteins with small diameters (GFP-Ana1 and GFP-Cep135). Note that the ninefold distributions of Ana1-GFP, Asl-GFP, GFP-Ana1, and GFP-Cep135 are well aligned with the N-terminus of Asl along the radial axes. Bars, 500 nm.

We then performed U-ExM to reveal the position of the Ana1 C-terminus in relation to the microtubule wall with higher resolution. The 3D-SIM image showed that the C-terminus of Ana1 was positioned between the microtubule blades (Fig. 3 A). Likewise, Asl-GFP was found in a similar pattern (Fig. 3 B). We asked whether Cep135, Ana1, and Asl are truly aligned along the radial axes.

**Figure S5.**
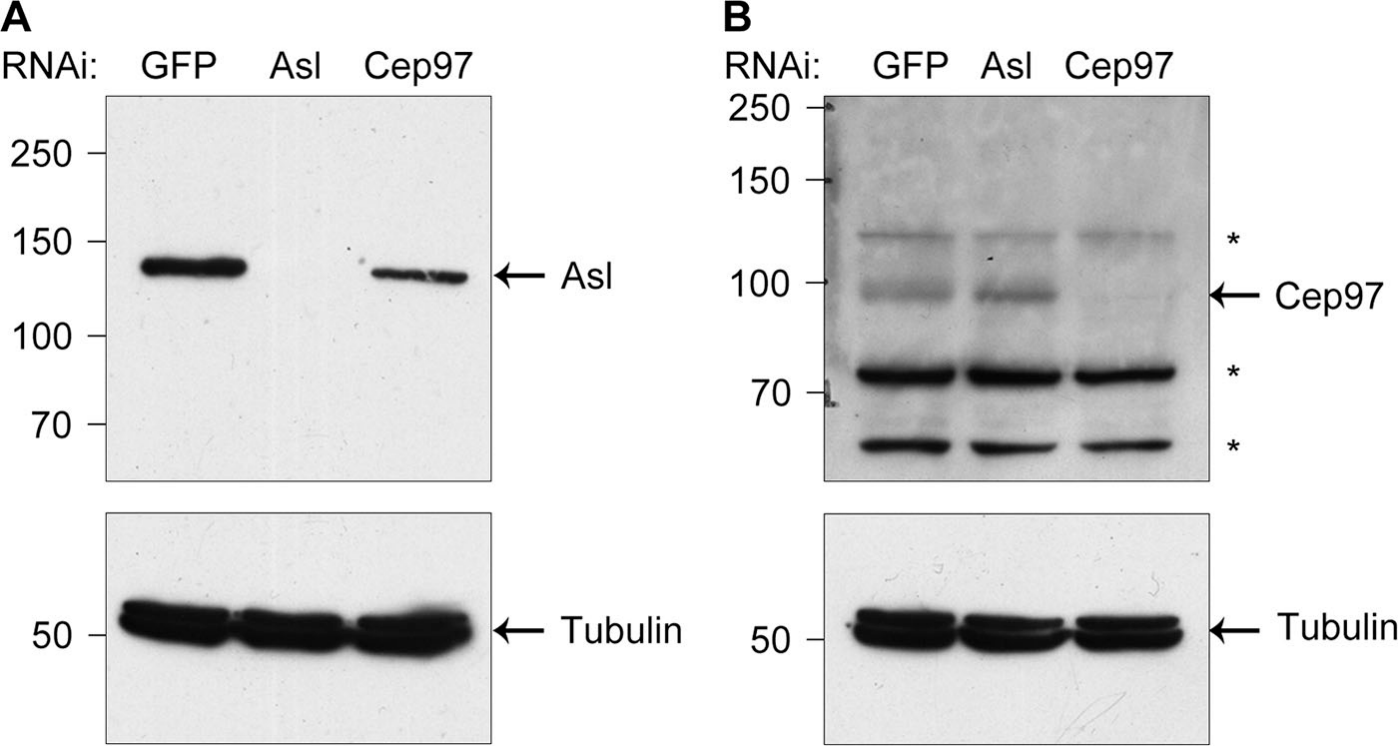
**Antibody verification. (A and B)** D.Mel-2 cells were depleted of GFP (control), endogenous Asl, or Cep97, and the whole-cell lysates were analyzed by Western blotting with Asl antibody (recognizes 1–300 aa; A) or Cep97 antibody (recognizes 670–806 aa; B). Tubulin serves as the loading control. *, nonspecific bands.

Deconvolved images were sufficient and of high-enough quality to analyze the N-terminus of Asl, Ana1-GFP, and Asl-GFP that formed large toroids, whereas reconstructed 3D-SIM images were required to analyze GFP-Ana1 and GFP-Cep135 that formed small toroids. We found that the ninefold symmetrical distributions of Ana1-GFP, Asl-GFP, GFP-Ana1, and GFP-Cep135 were all in line with the N-terminus of Asl (Fig. 3, C–F; and Fig. S5 A). These data show that the N-terminus of Cep135 and both termini of Ana1 and Asl are aligned along similar radial axes, which extend past the microtubule wall from between the blades.

### Decoration of Sas6-Cep135 axes by Ana2, Ana3, and Rcd4

**Figure 4.**
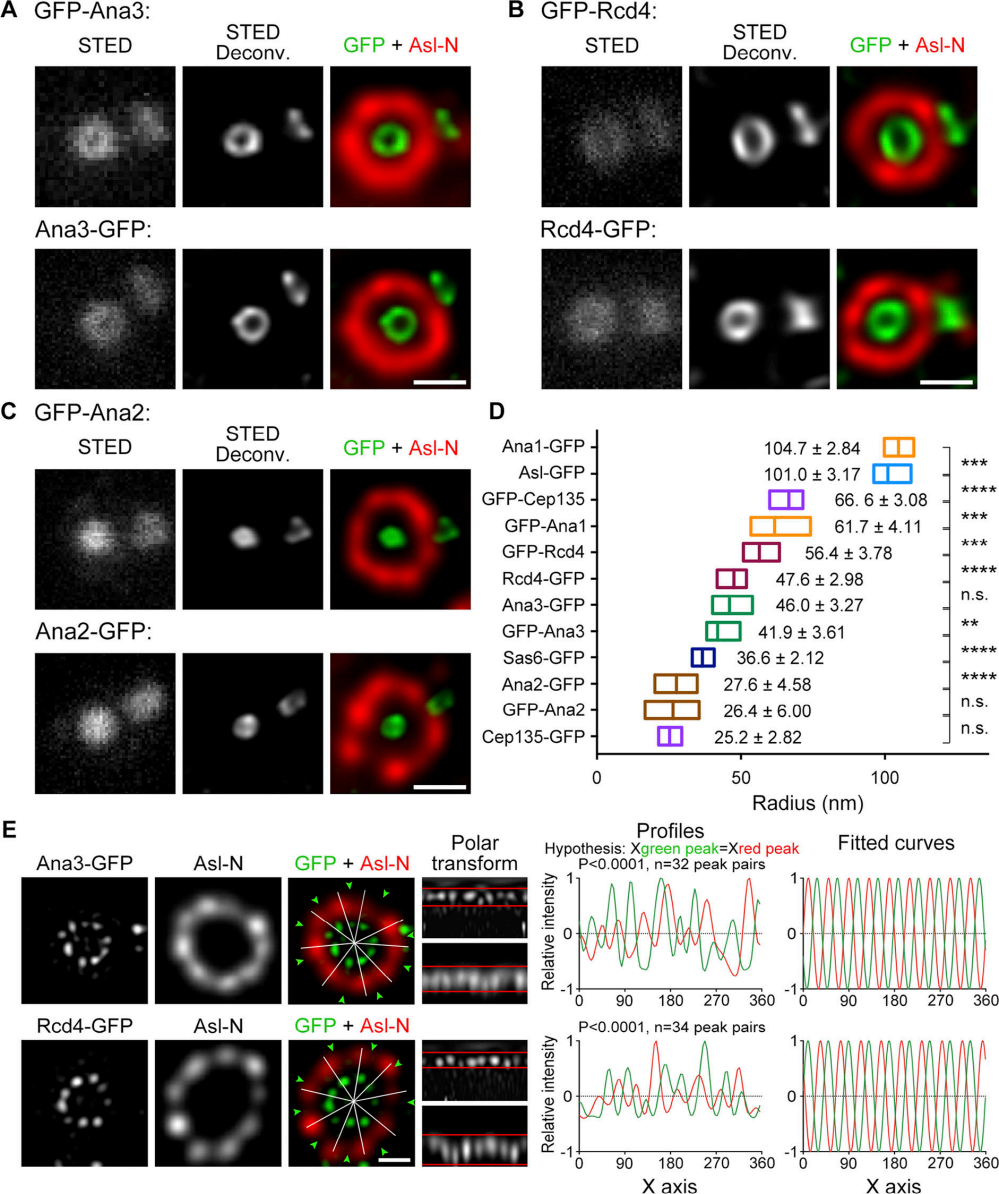
**Decoration of Sas6–Cep135 axes by Ana2, Ana3 and Rcd4. (A–C)** D.Mel-2 cells constitutively expressing GFP-tagged Ana3 (A), Rcd4 (B), or Ana2 (C) were immunostained with GFP-booster Atto647N (green) and antibody against the N-terminus of Asl (Asl-N; mother centriole marker, red) and analyzed by STED microscopy. Bars, 200 nm. **(D)** Mean radial distance of different regions of centriolar proteins. Horizontal low–high bar shows the range of the radius, and the vertical line indicates the mean. The mean radius ± SD is displayed next to each bar. ****, P < 0.0001 (unpaired, two-tailed Student’s *t* test); ***, P < 0.001; **, P < 0.01; n.s., not significant. From bottom up, *n* = 12, 20, 12, 20, 14, 23, 19, 19, 22, 16, 21, and 17 centrioles, respectively. **(E)** D.Mel-2 cells constitutively expressing Ana3-GFP or Rcd4-GFP were treated using the U-ExM protocol, immunostained with GFP (green) and Asl-N (recognizes 1–300 aa, red) antibodies, and imaged by 3D-SIM. Deconvolved images are used to analyze Asl-N and reconstructed 3D-SIM images for Ana3-GFP and Rcd4-GFP. Note that the ninefold distributions of Ana3-GFP and Rcd4-GFP are not in line with that of Asl-N. Bar, 500 nm.

The compact proteins included Ana3, Rcd4, and Ana2. Ana3 was reported to be responsible for the structural integrity of centrioles and basal bodies and for centriole cohesion in the *Drosophila* testes (Stevens et al., 2009). Rcd4 was identified to be involved in centriole duplication in a genome-wide RNAi screen (Dobbelaere et al., 2008). We found that both Ana3 and Rcd4 were core centriolar components localizing to the region that Cep135 occupied (Fig. 4, A, B, and D; and Fig. S2, G–J). Their N- and C-termini only showed marginal changes; however, we could not exclude the possibility that these proteins might expand their spatial occupation through domains inside the proteins. The N-terminus of Ana3 localized closest to the center of the centriole, followed by the C-termini of Ana3 and Rcd4 and the N-terminus of Rcd4. U-ExM revealed that the C-termini of Ana3 and Rcd4 were organized in a ninefold manner; moreover, the distributions of Ana3-GFP and Rcd4-GFP signals were not in line with the N-terminus of Asl, but rather had an obvious shift in the radial angle (Fig. 4 E). Among the proteins that we examined, the diameters of Ana2 and the C-terminus of Cep135 were at the borderline of the STED resolution. All signals can only be resolved into rings of ∼50-nm diameter after deconvolution processing (Fig. 2 B; Fig. 4, C and D; and Fig. S2, K and L).

### Ana3 and Rcd4 are distal to and partially overlap with Sas6

**Figure 5.**
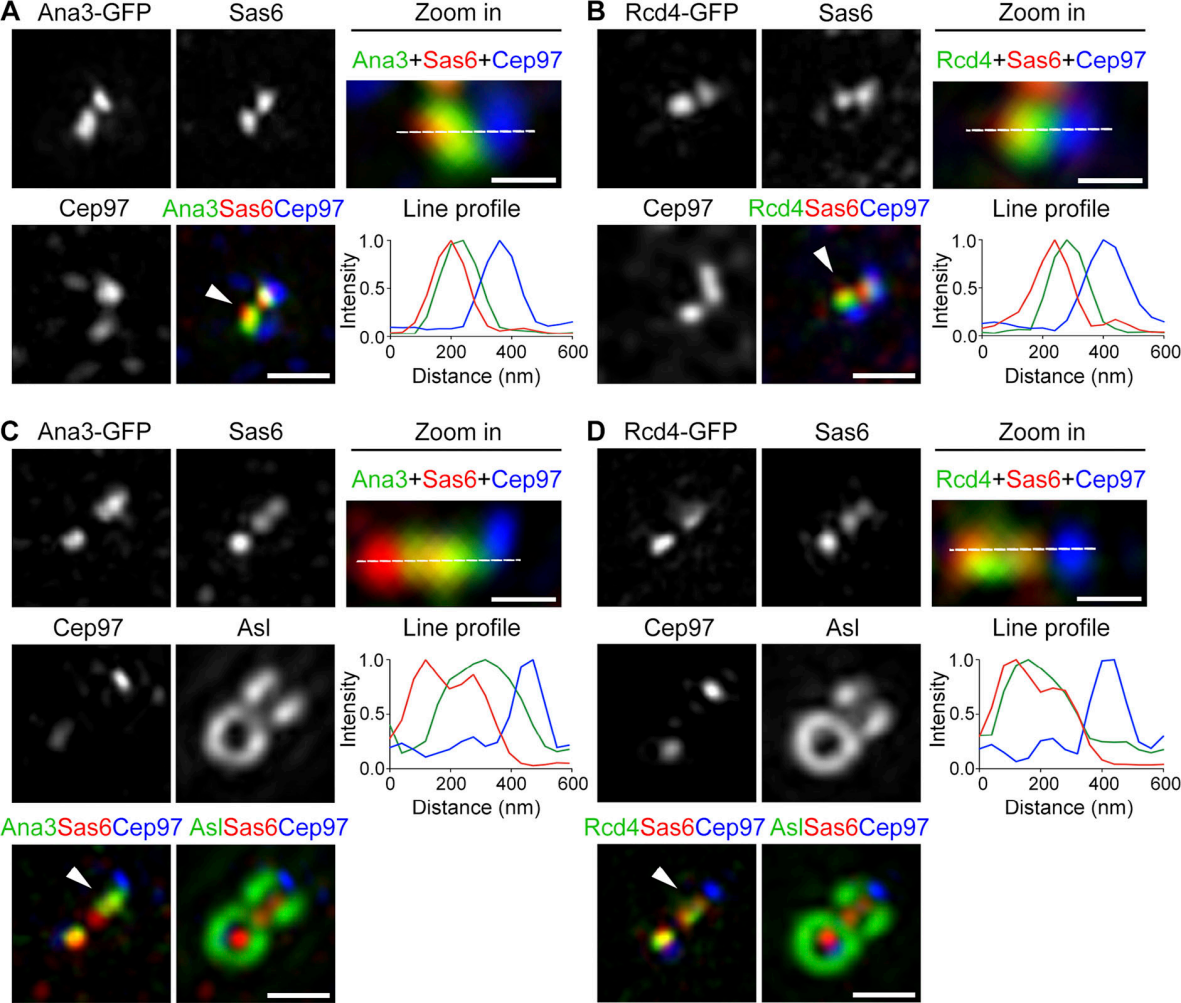
**Ana3 and Rcd4 are distal to and partially overlap with Sas6. (A and B)** D.Mel-2 cells constitutively expressing Ana3-GFP (A) or Rcd4-GFP (B) were immunostained with GFP-booster Atto488 (green) and antibodies against Sas6 (proximal marker, red) and Cep97 (distal marker, blue). 3D-SIM images revealed that Ana3 and Rcd4 largely overlap with Sas6, with their peak intensity shifting to the distal side of Sas6. Arrowheads mark the centrioles that are zoomed and measured. Fluorescence intensity along the dotted line drawn in each zoomed image is plotted as a function of the distance along the proximal-distal axis. Bars in left panels, 500 nm; for zoomed images, 200 nm. **(C and D)** *Drosophila* testes constitutively expressing Ana3-GFP (C) or Rcd4-GFP (D) were immunostained with GFP-booster Atto488 (green) and antibodies against Sas6 (proximal marker, red), Cep97 (distal marker, blue), and Asl (far red channel). 3D-SIM images revealed an extended distribution of Sas6 along the longitudinal axis of the centriole, and Sas6 partially overlaps with Ana3 and Rcd4. Arrowheads mark the centrioles that are zoomed and measured. Fluorescence intensity along the dotted line drawn in each zoomed image is plotted as a function of the distance along the proximal-distal axis. Bars in left panels, 500 nm; for zoomed images, 200 nm.

A previous study on the positions of RTTN and PPP1R35 (human counterparts of Ana3 and Rcd4, respectively) along the centriolar longitudinal axis reported that the two proteins localize to the proximal centriolar lumen above the cartwheel (Sydor et al., 2018). To test whether Ana3 and Rcd4 localize similarly, we costained D.Mel-2 cells constitutively expressing Ana3-GFP or Rcd4-GFP with GFP-booster Atto488, Sas6 (proximal marker), and Centrosomal protein 97kDa (Cep97; distal marker), and we examined their proximal–distal distributions. The centriole in D.Mel-2 cells is ∼175 nm long (Greenan et al., 2018), much shorter than the mammalian centriole (∼500 nm). Nevertheless, Sas6 and Cep97 signals were well separated along the centriolar proximal–distal axis (Fig. 5, A and B; and Fig. S5 B). The signals of Ana3 and Rcd4 were easily distinguished from that of Cep97, but they overlapped largely with Sas6. A line profile revealed that the peak intensity of Ana3 and Rcd4 shifted slightly to the distal side of Sas6. Our data were consistent with a recent study using the same cell line (Panda et al., 2020). Because the full widths at half maximum of the Ana3, Rcd4, and Sas6 signals were 43%, 35%, and 14% larger, respectively, than the point spread function (green channel, 104.166 nm; red channel, 117.349 nm), the overlapping signal could not be attributed only to the limit of resolution; rather, it suggests that Sas6 partially overlaps with Ana3 and Rcd4.

We also performed similar experiments using *Drosophila* spermatogonia cells where the centriole is composed of triplet microtubules and is longer than the D.Mel-2 centriole (Gottardo et al., 2015). We costained *Drosophila* testes constitutively expressing Ana3-GFP or Rcd4-GFP with GFP-booster Atto488, Sas6 (proximal marker), Cep97 (distal marker), and Asl, and we examined them using 3D-SIM. This revealed the slightly distal shift of Ana3 and Rcd4 signals compared with Sas6 and, again, obvious overlap between Ana3 and Sas6 and between Rcd4 and Sas6 (Fig. 5, C and D). These data corroborate the results from D.Mel-2 cells that Ana3 and Rcd4 are distal to and partially overlap with Sas6. It is thus possible that Ana3 and Rcd4 do not localize to the entire length of the cartwheel, which has recently been revealed to protrude proximally 10–40 nm beyond the microtubule wall in *Chlamydomonas*, *Paramecium*, *Naegleria*, and humans (Klena et al., 2020). It is also possible that, in addition to their cartwheel localization, Ana3 and Rcd4 further extend toward Cep97, the distal cap to the centriolar microtubule wall.

### Ana3 is recruited to the centriole after Sas6 and before Rcd4 and Cep135

**Figure 6.**
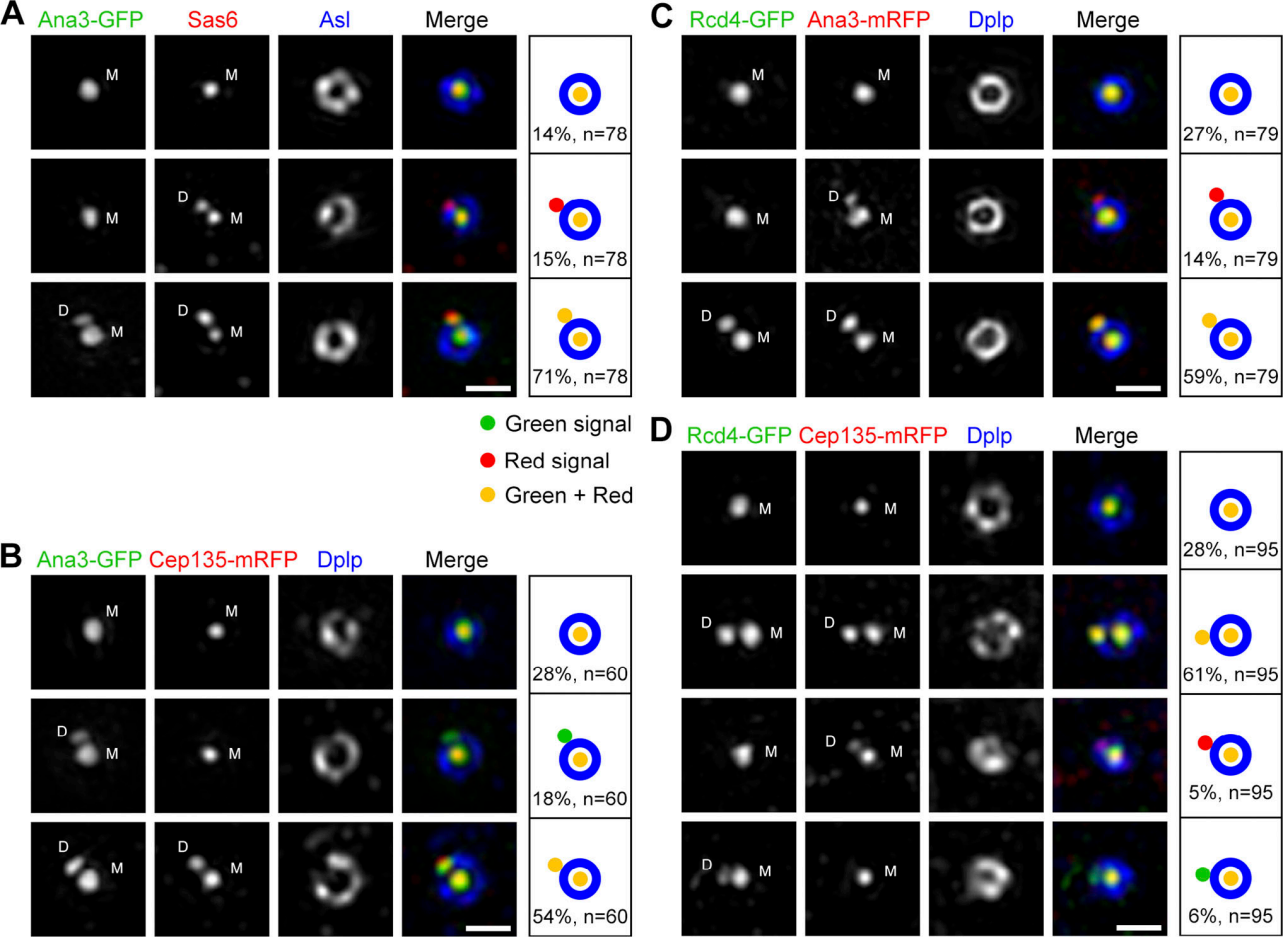
**Ana3 is recruited to the centriole after Sas6 and before Rcd4 and Cep135. (A)** D.Mel-2 cells constitutively expressing Ana3-GFP were immunostained with GFP-booster Atto488 (green), antibodies against Sas6 (red) and Asl (as mother centriole marker, blue), and DAPI (DNA staining, not shown). 3D-SIM images revealed that 15% (*n* = 78) of the interphase centrosomes have Sas6 signals at both mother and daughter centrioles, whereas Ana3 is only at the mother centriole, indicating Ana3 is recruited to the daughter centriole later than Sas6. M, mother centriole; D, daughter centriole. Bar, 500 nm. **(B–D)** D.Mel-2 cells constitutively expressing Ana3-GFP (B) or Rcd4-GFP (C and D) were transfected with indicated mRFP-tagged protein (red) and immunostained with GFP-booster Atto488 (green), Dplp antibody (as mother centriole marker, blue), and DAPI (not shown). 3D-SIM images revealed that Ana3 is recruited to the daughter centriole before Cep135 (B; 18% of interphase centrosomes, *n* = 60) and Rcd4 (C; 14% of interphase centrosomes, *n* = 79). Also note the simultaneous appearance of Rcd4 and Cep135 at the daughter centriole (D); no obvious hierarchy was observed between these two proteins (*n* = 95). Bars, 500 nm.

We sought to add a temporal resolution to these core centriolar proteins. Ana2 was reported to recruit Sas6 for initial centriole duplication upon phosphorylation by Plk4 (Dzhindzhev et al., 2014; Moyer et al., 2015; Ohta et al., 2014); Cep135, Ana1, and Asl are sequentially loaded onto the daughter centriole from late interphase to prophase for the centriole-to-centrosome conversion, the final stage in the assembly of the daughter centriole that converts it into a mother able to duplicate and recruit PCM (Fu and Glover, 2016; Fu et al., 2016). We costained D.Mel-2 cells constitutively expressing Ana3-GFP with Sas6 and Asl antibodies, and we performed 3D-SIM on the interphase centrosomes (Fig. 6 A). Three categories of centrioles were identified: 14% had a single dot of Sas6 and Ana3, indicating the daughter centriole had not formed; 71% had Sas6 and Ana3 at both mother and daughter centrioles; and 15% had Sas6 but no Ana3 at the daughter. Thus, Ana3 is recruited to the daughter centriole later than Sas6. Similar approaches discovered that the recruitment of Ana3 was always before Rcd4 and Cep135 (Fig. 6, B and C). We could not see obvious hierarchy between Rcd4 and Cep135; in most cases, both proteins were either absent or present at the daughter centriole (Fig. 6 D), indicating they might be recruited together or within a short time window. Of course, centrioles lacking a signal for a given protein could mean that it has not yet docked or that the protein is highly dynamic. However, the fact that we never observed Ana3 appearing before Sas6 at the daughter centriole or Rcd4 and Cep135 appearing before Ana3 indicates a timing order of the recruitment of Sas6, Ana3, and Rcd4/Cep135. We observed a small portion of daughter centrioles upon which Rcd4 and Cep135 were recruited without one another, and this suggests that the recruitment of both proteins might contribute to their stabilization at the centriole.

### Ana3 and Rcd4 are required for centriole-to-centrosome conversion but not for initial centriole duplication

**Figure 7.**
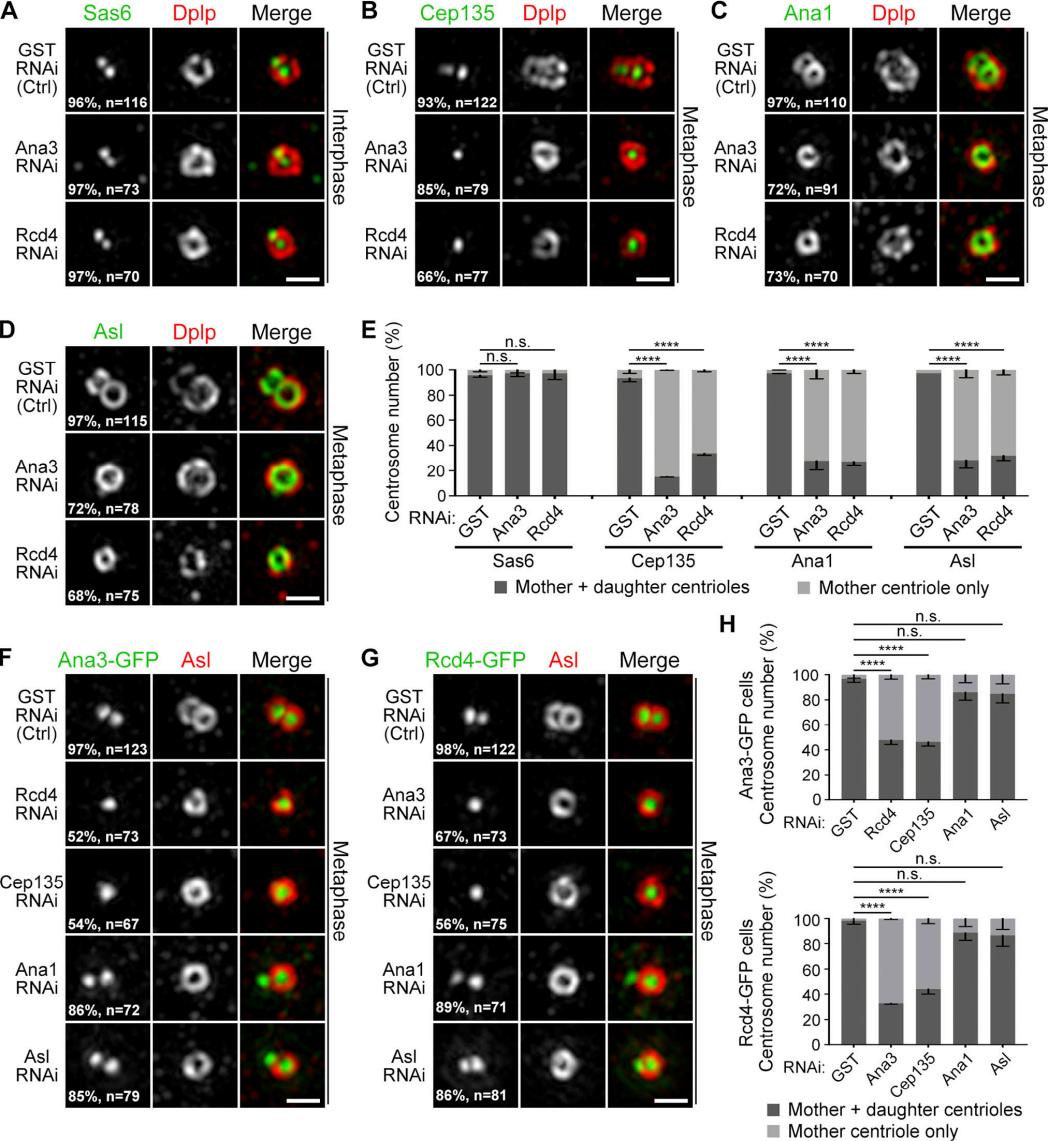
**Ana3 and Rcd4 are required for centriole-to-centrosome conversion but not for initial centriole duplication. (A)** D.Mel-2 cells were depleted of GST (control), endogenous Ana3, or Rcd4 and immunostained to reveal Sas6 (green), Dplp (mother centriole marker, red), and DNA (not shown). Cells with a single Dplp signal were imaged, indicating compromised centriole duplication. Almost all interphase centrosomes harbor Sas6 to a site for daughter centriole formation in control and depleted cells. n indicates the total centrosome number from three independent experiments. Bar, 500 nm. **(B–D)** D.Mel-2 cells were depleted of GST, endogenous Ana3, or Rcd4 and immunostained to reveal indicated proteins and phospho-histone H3 Ser10 (mitotic marker, not shown). Almost all metaphase centrosomes have Cep135 (B), Ana1 (C), and Asl (D) at daughter centrioles in control cells, whereas in Ana3- or Rcd4-depleted cells, a majority show absence of these three proteins from daughter centrioles. n indicates the total centrosome number from three independent experiments. Bars, 500 nm. **(E)** Quantification of protein recruitment at daughter centrioles in A–D. Error bars indicate SD. ****, P < 0.0001 (unpaired, two-tailed Student’s *t* test); n.s., not significant. **(F and G)** D.Mel-2 cells constitutively expressing Ana3-GFP (F) or Rcd4-GFP (G) were depleted of indicated protein. The localization of Ana3 and Rcd4 is affected by depletion of each other, of Cep135, but not by Ana1 or Asl. n indicates the total centrosome number from three independent experiments. Bars, 500 nm. **(H)** Quantification of protein recruitment at daughter centrioles in F and G. Error bars indicate SD. ****, P < 0.0001 (unpaired, two-tailed Student’s *t* test); n.s., not significant.

Given that the depletion of Ana3 or Rcd4 from cultured cells causes the reduction of the centrosome number (Dobbelaere et al., 2008; Goshima et al., 2007), we asked in which steps during centriole duplication they could play a role. We depleted endogenous Ana3 or Rcd4 from cells and examined the distributions of centriolar proteins at the daughter centriole. We selected cells with a single intact centrosome (Asl or *Drosophila* Pericentrin-like protein [Dplp] as a marker) indicating the impaired duplication cycle, and we found that the recruitment of Sas6 to the site for daughter centriole formation was not affected (Fig. 7, A and E). This suggests that the initial steps of the centriole duplication are not affected in the absence of Ana3 or Rcd4. On the contrary, in metaphase cells, >60% of the daughter centrioles failed to harbor Cep135, Ana1, and Asl (Fig. 7, B–E), the complex that should be associated with all daughter centrioles in this stage (Fu and Glover, 2016; Fu et al., 2016). This suggests that Ana3 and Rcd4 are required for the centriole-to-centrosome conversion through recruiting the Cep135–Ana1–Asl complex. Depletion of Ana3, Rcd4, or Cep135 affected the localization of the other two proteins at the daughter centriole (Fig. 7, B and E–H). Thus, the three proteins are interdependent for their centriolar localization, which is consistent with their overlapping spatial distributions at the centriole core. Not surprisingly, the recruitment of Ana3 and Rcd4 to the daughter centriole was not significantly affected by the depletion of Ana1 or Asl, as long as the initial assembly of the daughter centriole was not compromised (indicated by positive staining of Asl at the mother centriole; Fig. 7, F–H).

## Discussion

**Figure 8.**
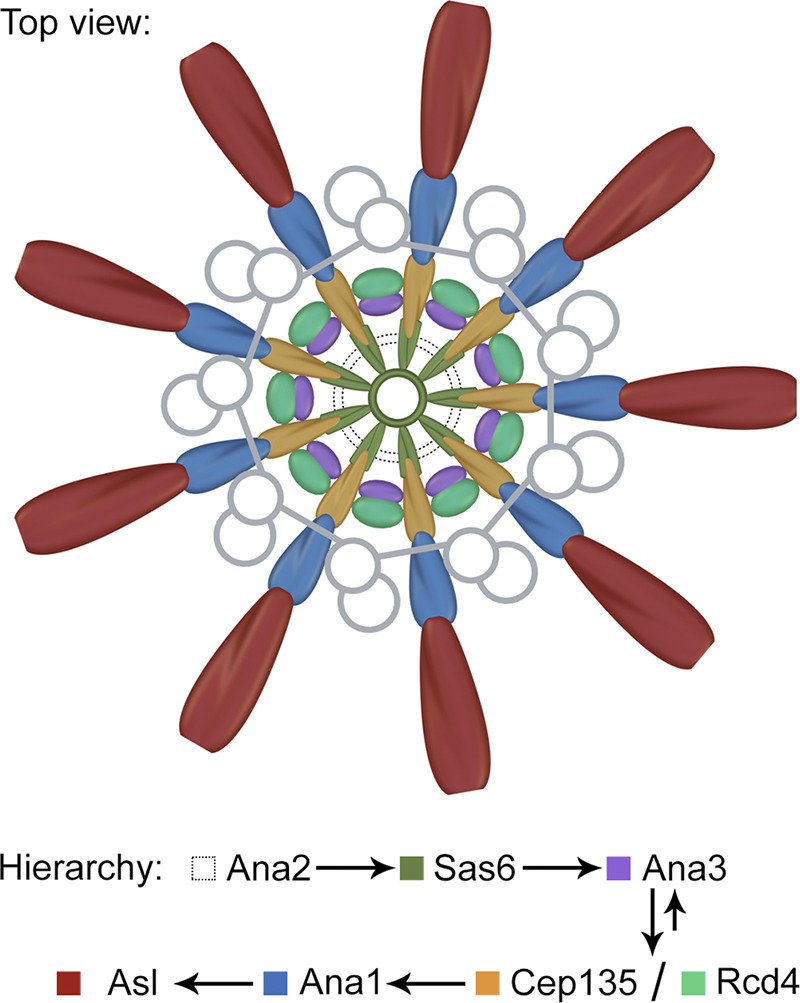
**Schematics depicting the lateral organization of centriole core.** Cep135, Ana1, and Asl are each organized in ninefold symmetry aligned with one another. Together with Sas6, they constitute the nine radial axes extending past the centriole microtubule wall from between the blades. Ana2, Ana3, and Rcd4 are a group of compact proteins possibly supporting these radial axes, with Ana3 and Rcd4 organized also in ninefold symmetry that does not match the above axes. Arrows indicate the hierarchy of these proteins. Ana3 is recruited to the centriole before Rcd4 and Cep135, while all three proteins are interdependent for their centriolar localization.

Here our data reveal the spatiotemporal organization of the proteins at the core region of the *Drosophila* centriole (Fig. 8). By superimposing our measurements to the electron cryotomography data of the *Trichonympha*, *Chlamydomonas*, and *Drosophila* centrioles (Greenan et al., 2018; Guichard et al., 2013; Guichard et al., 2017), we found that Cep135 overlaps with the C-terminus of Sas6 on the spokes via its C-terminus and extends to the pinheads via the N-terminus. Ana1 localizes from the pinheads to the outer edge of the doublet microtubules. Asl slightly overlaps with the doublet microtubules and extends into PCM in a ninefold manner. We propose that the core region of the centriole is composed of two dimensions. One is the ninefold radial dimension that is established by elongated molecules overlapping through their adjacent termini: Sas6, Cep135, Ana1, and Asl. They likely constitute the spoke–pinhead axes and further transmit the ninefold symmetrical geometry to the microtubule wall and into the core PCM. The other is a circular dimension established by a group of compact proteins that are also arranged in ninefold symmetry: Ana3, Rcd4, and possibly Ana2 (Qiao et al., 2012). They likely decorate the radial axes and provide the physical support for the ninefold configuration.

We previously showed that Cep135, Ana1, and Asl form a complex that is responsible for the centriole-to-centrosome conversion (Fu and Glover, 2016; Fu et al., 2016), the final stage in the assembly of the daughter centriole that converts it into a mother centriole able to duplicate (Wang et al., 2011). Here, with improved spatial resolution, we show that the three proteins are each organized in ninefold manner (Fig. 1 B; and Fig. 2, D and E), reinforcing the idea they are the bona fide components of the spoke–pinhead scaffold. The ninefold radial axes then extend past the centriole microtubule wall via the C-terminus of Ana1, which is positioned between the microtubule blades (Fig. 3). Recently, an electron cryotomography study showed that, between adjacent microtubule blades, there are ninefold amorphous brushlike structures in the *Drosophila* S2 centriole (Greenan et al., 2018). Our study suggests that it could contain Ana1 and Asl, both of which exhibit ninefold symmetry at this region.

Our findings allocate a role to *Drosophila* Ana3 and Rcd4, previously known from genome-wide RNAi screens to be required for centriole duplication (Dobbelaere et al., 2008; Goshima et al., 2007). Ana3 was later reported to be responsible for the structural integrity of centrioles and basal bodies and for centriole cohesion in the *Drosophila* testes (Stevens et al., 2009). We now provide evidence that both Ana3 and Rcd4 are core centriolar components, localizing to the region where Cep135 is (Fig. 4, A, B, and D). The N-terminus of Ana3 localizes closest to the center of the centriole, followed by the C-termini of Ana3 and Rcd4 and the N-terminus of Rcd4. Both Ana3 and Rcd4 are organized in ninefold symmetry but seem to be positioned in axes that are not in line with the Cep135–Ana1–Asl complex (Fig. 4 E). Spatial overlapping of Ana3 and Rcd4 indicates these two proteins might interact, which has recently been reported (Panda et al., 2020) and is conserved to their human counterparts, RTTN and PPP1R35 (Sydor et al., 2018). Depletion of either Ana3 or Rcd4 leads to failure in loading the Cep135–Ana1–Asl complex during centriole biogenesis (Fig. 7) and thus causes defects in centriole-to-centrosome conversion and the reduction of the centrosome number. This pathway is also conserved in human cells, where PPP1R35 was reported to promote centriole-to-centrosome conversion upstream of Cep295 (human homologue of Ana1; Fong et al., 2018) and RTTN and PPP1R35 serve as upstream effectors of Cep295 in mediating centriole elongation (Chen et al., 2017; Sydor et al., 2018).

Taken together, our data provide an overall picture of the protein architecture at the centriole core and implications of how the ninefold symmetrical structure might be built. Knowing the spatiotemporal restraints of individual centriolar components will guide the immediate study of the molecular interaction partners and understanding of their functions. Meanwhile, it would also provide information for a higher-resolution approach, including cryo-EM, to eventually obtain a 3D map of the centriole.

## Materials and methods

### DNA constructs

cDNA clones for *sas6* (AT29216), *ana2* (LD22033), *cep135* (LD35990), *ana1* (LD07765 and IP16240), and *asl* (GH02902) were obtained from the *Drosophila* Genomics Resource Center (DGRC) and previously described (Dzhindzhev et al., 2014; Fu et al., 2016). The cDNA clone for *rcd4* (SD16838) was obtained from DGRC, and full-length *ana3* was amplified from the genomic DNA of Oregon R flies. Entry clones with the above coding sequence were generated using the Gateway System (Invitrogen/Thermo Fisher Scientific). Expression constructs were made by recombination between entry clones and the following destination vectors: pAGW or pAWG (for actin 5C promoter-driven N- or C-terminal GFP fusion), pUGW or pUWG (for polyubiquitin promoter-driven N- or C-terminal GFP fusion), and pAWR (for actin 5C promoter-driven C-terminal mRFP fusion), all from DGRC; pattB-pUWG (constructed in-house to generate transgenic flies at a predetermined genome location; the multiple cloning site on the pattB vector [http://www.flyc31.org/] was replaced by a polyubiquitin promoter, Gateway cassette, EGFP, triple stop, and Hsp27 terminator from pUWG).

### Cell culture, transfection, stable cell lines, and RNAi

D.Mel-2 cells were grown at 25°C in Express Five SFM (Gibco/Thermo Fisher Scientific) supplemented with L-glutamine (2 mM; Gibco) and penicillin-streptomycin (50 U/ml, 50 µg/ml; Gibco) and checked regularly to ensure they were mycoplasma free. Transfection of plasmids was performed using X-tremeGENE HP DNA Transfection Reagent (Roche). To establish stable cell lines, an additional plasmid carrying blasticidin resistance was cotransfected, and 20 µg/ml blasticidin (Gibco) was added to the medium 48 h after transfection. Established cell lines were authenticated by both immunostaining and PCR amplification of the transgenes. To perform RNAi experiments, cells were transfected with double-stranded RNA by using TransFast Transfection Reagent (Promega) and collected after 4 d (e.g., Ana3, Ana1, and Asl). For repeated rounds of depletion, cells were collected every 4 d and resubmitted to the same transfection protocol (e.g., Cep135 and Rcd4, three rounds of depletion; Cep97, two rounds of depletion). RNAi efficiency was tested by Western blotting, and cells with a single Dplp or Asl signal were imaged, indicating compromised centriole duplication. Double-stranded RNA directed against the coding sequence was synthesized from cDNA template using the T7 RiboMAX Express RNAi System (Promega), and the primers used were as previously described (Dobbelaere et al., 2008; Fu et al., 2016).

### Fly stocks

Fly stocks were maintained at 25°C on standard *Drosophila* food. *Polyubiquitin Rcd4-GFP/TM6B* transgenic flies were generated by the Tsinghua Fly Center, and *polyubiquitin Ana3-GFP/Cyo* was provided by Jordan Raff (University of Oxford, Oxford, UK; Stevens et al., 2009).

### Antibodies

The following antibodies were used: rabbit anti-Asl (1:500, recognizes the N-terminus of the protein; Dzhindzhev et al., 2010; Fu and Glover, 2012), chicken anti-Dplp (1:500; Rodrigues-Martins et al., 2007), rat anti-Sas6 (1:500, against GST-Sas6-236-472 aa; Dzhindzhev et al., 2014), rabbit anti-Ana1 (1:500, against His-Ana1-1400-1729 aa; Fu et al., 2016), guinea pig anti-Cep135 (1:500, against His-Cep135-810-1059 aa), rabbit anti-GFP (1:500; Fu et al., 2009), guinea pig or rabbit anti-Asl and rabbit anti-Cep97 (1:500, against His-Asl-1-300 aa and GST-Cep97-670-806 aa, respectively; serum produced by the Animal Facility, Institute of Genetics and Developmental Biology, Chinese Academy of Sciences, and purified as previously described; Fu et al., 2016), mouse anti-acetylated tubulin (1:500, T7451; Sigma-Aldrich), mouse anti–phospho-histone H3 Ser10 (1:250, 9706; Cell Signaling Technology), GFP-booster Atto488 (1:200, ChromoTek), GFP-booster Atto647N (1:200, ChromoTek), and HRP-conjugated anti–β-tubulin (1:5,000, BE3312; Shenzhen Bioeasy Biotechnology). Secondary antibodies were conjugated with Alexa Fluor 405, 488, 568, or 647 (1:500; Invitrogen), with Abberior STAR RED (1:150; Abberior), and with HRP (1:10,000; Jackson ImmunoResearch). The fluorescent nanoparticles were from Abberior (1× Nanoparticles Red Fluor, 40 nm; NP-3004).

### Immunofluorescence

D.Mel-2 cells were plated on Con A (Sigma-Aldrich)-coated coverslips (no. 1.5; 0.17 mm thick; Zeiss) 3 h before fixation. Cells were washed once with PBS and fixed with precooled methanol for 6 min at −20°C. After rehydration in PBS, the cells were incubated with GFP-booster and/or the primary antibody overnight at 4°C and subsequently washed and incubated with the secondary antibody for 45 min at RT. Coverslips were mounted onto slides using VECTASHIELD antifade mounting medium (VECTOR Laboratories; H-1000-10) for 3D-SIM imaging or using Mowiol 4-88 (Sigma-Aldrich) mounting medium for STED imaging. To view anti-acetylated tubulin–stained centrioles, cells were first treated with 1 µg/ml colchicine (Sigma-Aldrich) for 12 h, prefixed with 1% PFA containing 0.5% Triton X-100 for 2 min, and fixed with 4% PFA containing 0.5% Triton X-100 for 15 min at RT. Cells were blocked with 3% BSA/PBS for 30 min before incubation with antibodies.

*Drosophila* testes from third-instar larvae or pharate adults were dissected in PBS, transferred to 5% glycerol/PBS, and squashed between a microscope slide and coverslip. After snap freezing in liquid nitrogen, testes on slides were fixed in methanol, rehydrated in 0.5% Triton X-100/PBS for 30 s, rinsed in PBS for 10 min, and incubated with primary antibodies (diluted in PBS) at 4°C overnight. Slides were then rinsed for 30 s in PBS and incubated again in PBS for 10 min and with secondary antibodies (1:200 in PBS) for 4 h at RT. Finally, slides were rinsed in PBS for 30 s followed by a 10-min wash and mounted in VECTASHIELD antifade mounting media (Vector Laboratories; H-1000-10).

### U-ExM

U-ExM was performed as previously reported (Gambarotto et al., 2019). Briefly, D.Mel-2 cells were plated on Con A (Sigma-Aldrich)-coated coverslips and incubated in a PBS solution containing 1.4% formaldehyde (Sigma-Aldrich; F8775) and 2% acrylamide (Sigma-Aldrich; A4058) for 5 h at RT. Next, coverslips with cells facing down were incubated with U-ExM monomer solution composed of 19% (wt/wt) sodium acrylate (Sigma-Aldrich; 408220), 10% acrylamide, 0.1% *N,N'*-methylenebisacrylamide solution (Sigma-Aldrich; M1533), 0.5% ammonium persulfate (Sigma-Aldrich; V900883), and 0.5% *N,N,N',N'*-tetramethylethylenediamine (Sigma-Aldrich; V900853). Gelation was performed on ice for 5 min and at 37°C for 1 h in a humidified chamber. Gels were then transferred from coverslips into the denaturation buffer (200 mM SDS, V900859; Sigma-Aldrich; 200 mM NaCl, V900058; Sigma-Aldrich; 50 mM Tris, pH 9.0; V900483; Sigma-Aldrich) at 95°C for 1.5 h and expanded for the first round in double-distilled H_2_O. Gels were labeled with primary and secondary antibodies for 3 h at 37°C, respectively. Prior to 3D-SIM imaging, gels were fully expanded and placed on poly-L-lysine (Sigma-Aldrich; P4707)-coated coverslips.

### Superresolution imaging

The procedures of 3D-SIM were previously described (Fu and Glover, 2012; Fu et al., 2016). Briefly, superresolved images were acquired using a DeltaVision OMX SR imaging system (GE Healthcare) equipped with four scientific complementary metal-oxide semiconductor cameras; 405-, 488-, 568-, and 647-nm laser illumination; an Olympus plan apochromat N 60× 1.42 NA oil objective; and standard excitation and emission filter sets. Raw data were collected at RT using three angles and five phase shifts of the illumination pattern in AcquireSR software (GE Healthcare). The data were then reconstructed to get superresolved 3D-SIM images or deconvolved to get widefield/deconvolved images by using DeltaVision softWoRx software (GE Healthcare). Reconstruction was performed using channel-specific optical transfer functions, a Wiener filter of 0.001, and channel-specific K0 angles. Deconvolution was performed using the enhanced ratio method, 10 cycles, and medium noise filtering (200 nm). The refractive index of the immersion oil (Cargille Laboratories) was adjusted to minimize spherical aberrations. Sections were acquired at 0.125-µm z steps.

STED imaging was performed at RT on a Leica TCS SP8 STED 3X microscope equipped with an HyD single-molecule detection hybrid detector; pulsed white light laser illumination; multiple STED laser lines at 592 nm, 660 nm, and the pulsed laser at 775 nm; a high-contrast plan apochromat 100×, 1.40 NA oil CS2 objective; and Application Suite X software (LAS X; Leica Microsystems). The power of the depletion laser was set differently for different proteins on the basis of photon counts. Emitted fluorescence was filtered with a confocal pinhole of 1.0 Airy unit, and only photons with a lifetime between 0.5 and 10.0 ns were collected (LightGate). Huygens Professional software (Scientific Volume Imaging) was used for post-processing deconvolution of raw STED images. The optimized iteration mode of the classic maximum likelihood estimation was applied until it reached a quality threshold of 0.001 or a maximum of 40 iterations. The signal-to-noise ratio was set to 20 for GFP signals and 15 for the N-terminus of Asl and acetylated tubulin signals.

### Data processing and statistical analysis

To better measure the diameter of the protein distribution, centrioles perpendicular to the coverslips were selected before further analysis. A reported method was adopted (https://github.com/MicronOxford/cool; Gartenmann et al., 2017). Briefly, each centriole was cropped from the full-sized STED image. An initial guess was calculated and fed to an elliptical annular generator. The generator then took eight parameters (center_x, center_y, radius_x, radius_y, angle, width, amplitude, and background) to create a simulated centriole. To obtain the best-fitting parameters where the mean square error between two images reaches a minimum, a least-squares with Levenberg-Marquardt algorithm was performed. It would iterate until each step of parameters satisfied tolerance of termination. Last, the eccentricity (major/minor semiaxis ratio) of each centriole was calculated, and the centrioles with numbers between 1 and 1.2 were considered to be upright. All analyses were performed using Asl signal (the reference channel) for consistency.

The following image processing and analyses were performed in MATLAB. To get the radius of the protein distribution in Figs. 2 C and 4 D, a two-stage fitting strategy was adopted. First, a ring-shape pattern was detected by an adaptive threshold (mean + β × SD; β was determined by the type of centriole proteins), and pixels above threshold were fitted by Kåsa’s method that is based on least squares. Next, on the basis of the coarse fitting result, a derivative-free optimization (fminsearch) was implemented to search for the circular profile (center coordinates and radius) where the average intensity on the fitted ring reached the maximum. The mean radius and standard deviation for each protein were then calculated using GraphPad Prism 5 software.

To determine if a centriolar protein is organized in ninefold symmetrical distribution, a peak-to-peak angle analysis was performed. First, the toroid in each image was equally divided into 256 sectors, and average intensity of each sector (angular intensity) was calculated. Then a bandpass Fourier filter was used to reduce the noise. The filtered angular intensity was plotted, and peaks were determined by using a nonmaximum suppression algorithm that retained the main peaks while removing the subpeaks whose value was unequal to the maximum of neighborhood. The mean angle and standard deviation were calculated using GraphPad Prism 5 software.

To compare angular distributions of two centriolar proteins or termini, U-ExM images were remapped to polar coordinates (polar transform) where the center of the centriole was located by a similar method used for STED images and the angular step was fixed to 4°. Signals within the selected rectangle were plotted along the horizontal axis to generate the radial profiles, which were further fitted to sine waves with fixed frequency of 9. The phase difference between two compared waves represents the relative distributions of two proteins along their angular axis.

### Online supplemental material

Fig. S1 shows the optimization of the STED microscopy setup, where the combination of pulsed STED laser and time-gated detection renders the best resolution for the centriolar proteins. Fig. S2 lists two representative STED images for each centriolar protein examined as a supplement to the main figures. Fig. S3 shows that the N-terminus of Sas6 is resolved as a dot in either a raw or deconvolved STED image. Fig. S4 shows the C-terminus of Ana1 colocalizes with the microtubule wall under STED resolution. Fig. S5 presents the verification of the antibodies.
